# HBeAg Levels Vary across the Different Stages of HBV Infection According to the Extent of Immunological Pressure and Are Associated with Therapeutic Outcome in the Setting of Immunosuppression-Driven HBV Reactivation

**DOI:** 10.3390/biomedicines9101352

**Published:** 2021-09-29

**Authors:** Lorenzo Piermatteo, Mohammad Alkhatib, Stefano D’Anna, Vincenzo Malagnino, Ada Bertoli, Eleonora Andreassi, Elisa Basile, Alessandra Iuvara, Maria De Cristofaro, Giuseppina Cappiello, Carlotta Cerva, Carmine Minichini, Mariantonietta Pisaturo, Mario Starace, Nicola Coppola, Carla Fontana, Sandro Grelli, Francesca Ceccherini-Silberstein, Massimo Andreoni, Upkar S. Gill, Patrick T. F. Kennedy, Loredana Sarmati, Romina Salpini, Valentina Svicher

**Affiliations:** 1 Department of Experimental Medicine, University of Rome Tor Vergata, 00133 Rome, Italy; lorenzo.piermatteo@uniroma2.it (L.P.); mohammad--alkhatib@hotmail.com (M.A.); stefanodanna26@gmail.com (S.D.); bertoli@uniroma2.it (A.B.); eleoand@gmail.com (E.A.); grelli@med.uniroma2.it (S.G.); ceccherini@med.uniroma2.it (F.C.-S.); valentina.svicher@uniroma2.it (V.S.); 2 Infectious Disease Unit, University Hospital of Rome Tor Vergata, 00133 Rome, Italy; malagninovincenzo@gmail.com (V.M.); carlottacerva@gmail.com (C.C.); andreoni@uniroma2.it (M.A.); sarmati@med.uniroma2.it (L.S.); 3 Microbiology and Virology Unit, University Hospital of Rome Tor Vergata, 00133 Rome, Italy; elisa.basile@ptvonline.it (E.B.); alessandra.iuvara@ptvonline.it (A.I.); carla.fontana@uniroma2.it (C.F.); 4 Microbiology Unit, “Sandro Pertini” Hospital, 00133 Rome, Italy; maria.decristofaro@aslroma2.it (M.D.C.); giuseppina.cappiello@aslroma2.it (G.C.); 5 Department of Medical, Surgical, Neurological, Metabolic and Aging Sciences, University of Campania Luigi Vanvitelli, 80138 Naples, Italy; carmine.minichini@alice.it (C.M.); mariantonietta.pisaturo@unicampania.it (M.P.); mariostarace1984@libero.it (M.S.); nicola.coppola@unicampania.it (N.C.); 6 Barts Liver Centre, Immunobiology, Blizard Institute, Barts and the London School of Medicine and Dentistry, Queen Mary University of London, London E1 2AT, UK; u.gill@qmul.ac.uk (U.S.G.); p.kennedy@qmul.ac.uk (P.T.F.K.)

**Keywords:** HBV, HBeAg, HBV reactivation

## Abstract

HBeAg is a marker of HBV-activity, and HBeAg-loss predicts a favorable clinical outcome. Here, we characterize HBeAg-levels across different phases of HBV infection, their correlation with virological/biochemical markers and the virological response to anti-HBV therapy. Quantitative HBeAg (qHBeAg, DiaSorin) is assessed in 101 HBeAg+ patients: 20 with acute-infection, 20 with chronic infection, 32 with chronic hepatitis and 29 with immunosuppression-driven HBV-reactivation (HBV-R). A total of 15/29 patients with HBV-R are monitored for >12 months after starting TDF/ETV. qHBeAg is higher in immunosuppression-driven HBV-R (median[IQR]:930[206–1945]PEIU/mL) and declines in chronic hepatitis (481[28–1393]PEIU/mL, *p* = 0.03), suggesting HBeAg production, modulated by the extent of immunological pressure. This is reinforced by the negative correlation between qHBeAg and ALT in acute infection (Rho = −0.66, *p* = 0.006) and chronic hepatitis (Rho = −0.35; *p* = 0.05). Interestingly, qHBeAg strongly and positively correlates with qHBsAg across the study groups, suggesting cccDNA as a major source of both proteins in the setting of HBeAg positivity (with limited contribution of integrated HBV-DNA to HBsAg production). Focusing on 15 patients with HBV-R starting TDF/ETV, virological suppression and HBeAg-loss are achieved in 60% and 53.3%. Notably, the combination of qHBeAg > 2000 PEIU/mL + qHBsAg > 52,000 IU/mL at HBV-R is the only factor predicting no HBeAg loss (HBeAg loss: 0% with vs. 72.7% without qHBeAg > 2000 PEIU/mL + qHBsAg > 52,000 IU/mL, *p* = 0.03). In conclusion, qHBeAg varies over the natural course of HBV infection, according to the extent of immunological pressure. In the setting of HBV-R, qHBeAg could be useful in predicting the treatment response under immunosuppression.

## 1. Introduction

Hepatitis B virus (HBV) is a major global health problem and a leading cause of death. According to recent WHO estimates, 257 million people have a chronic HBV infection, resulting in 800,000 deaths every year, attributed to cirrhosis and liver cancer [1].

Among HBV proteins, the hepatitis B ‘e’ antigen (HBeAg) is not a structural protein since it is released as a soluble protein from the infected hepatocytes [2,3,4]. HBeAg is not essential for HBV transmission and replication [3,5]. Nevertheless, HBeAg can play a role in viral persistence, promoting chronicity through modulation of the immune response [6,7]. In particular, some studies suggested that HBeAg can have tolerogenic effects that dampen the innate and adaptive HBV immune response [5].

In clinical practice, HBeAg is an important biomarker whose qualitative detection in the serum of HBV-infected patients is usually indicative of intensive HBV replicative activity. In particular, HBeAg can be detected in acute infection, in patients undergoing immunosuppression-driven HBV reactivation and during the first two phases of chronic infection, named HBeAg-positive infection and HBeAg-positive chronic hepatitis, respectively. HBeAg-positive chronic infection is characterized by elevated serum HBV-DNA coupled with normal transaminases and no liver disease. This phase is then followed by HBeAg-positive chronic hepatitis, characterized by elevated ALT and reduction in serum HBV-DNA, reflecting the activation of an antiviral immune response that can constrain viral replication [8]. During the natural history of chronic HBV infection, the spontaneous or treatment-induced achievement of HBeAg loss is considered a clinically relevant endpoint associated with a favorable long-term outcome [8,9,10].

Immunosuppression-driven HBV reactivation is a clinically relevant topic that can lead to severe forms of liver injury [11]. Treatment with highly potent anti-HBV-drugs (entecavir or tenofovir) is recommended in patients undergoing HBV reactivation [11]. Nevertheless, very limited information is available on the kinetics of virological responses to anti-HBV drugs under iatrogenic immune suppression [12] and the role of HBV biomarkers in predicting HBeAg loss in this setting.

In previous years, homemade assays for the quantitative determination of HBeAg (qHBeAg) have been developed and used to explore the role of qHBeAg in predicting the achievement of HBeAg loss during anti-HBV treatment [13,14,15,16,17,18,19,20,21]. In particular, previous studies have highlighted that the on-treatment decline of qHBeAg can help to distinguish between slow, rapid and non-responders to nucleoside/nucleotide analogue therapy and also to identify patients most likely to experience virological breakthrough [18,19,20,21]. Similarly, on-treatment monitoring of qHBeAg may help to predict the likelihood of subsequent HBeAg seroconversion in patients treated with pegylated interferon [14,16,17].

To date, standardized assays for HBeAg quantification have been developed that will permit to better unravel the biological significance and clinical phenotype of qHBeAg in the setting of HBV infection.

In this light, by using a commercially available assay for HBeAg quantification, this study is aimed at characterizing (i) HBeAg levels across different phases of HBV infection, (ii) their correlation with virological and biochemical markers, and (iii) their role in predicting the virological response to anti-HBV therapy, particularly under iatrogenic immunosuppression.

## 2. Materials and Methods

### 2.1. Study Population

This retrospective study included 101 patients, monitored in the following clinical centers of Central/Southern Italy and in the U.K.: University Hospital Tor Vergata (Rome, Italy), “Sandro Pertini” Hospital (Rome, Italy), University Hospital of Campania “Luigi Vanvitelli” (Naples, Italy) and the Barts Liver Centre (London, UK). Inclusion criteria were as follows: HBsAg positivity, HBeAg positivity, being treatment naïve, and the availability of a plasma sample (residual from clinical practice) in a sufficient amount for the quantification of HBeAg and other markers of HBV replication. In particular, plasma samples were collected in a time window from 2010 to 2021. Exclusion criteria were as follows: pregnancy and concomitancy of hepatitis C or D virus, human immunodeficiency virus infection or a coexistent autoimmune/metabolic liver disease.

In particular, patients were classified as follows:

- 20 patients with acute HBV infection;
- 20 patients with HBeAg-positive chronic infection;
- 32 patients with HBeAg-positive chronic hepatitis (with elevated ALT);
- 29 patients undergoing immunosuppression-driven HBV reactivation.

The definition of acute HBV infection was based on the positivity to HBsAg and serum HBV DNA, positivity to anti-HBV core antigen immunoglobulin M (anti-HBc IgM) and altered alanine aminotransferase levels. All patients had also a clinical history compatible with acute HBV infection. After the diagnosis of acute HBV infection, patients were monitored for at least 12 months with a frequency based on the severity of clinical manifestation and on the internal protocols specific for each clinical center. In particular, during monitoring, the following parameters were collected in order to assess the outcome of HBV infection (clinical resolution/chronicity): serum HBV-DNA, transaminases and qualitative detection of HBsAg and HBeAg. HBeAg quantification was carried out only in the plasma samples at the diagnosis of acute infection in order to establish the correlation with levels of HBsAg, serum HBV-DNA and ALT observed at the diagnosis of acute infection and to evaluate the potential relationship with the outcome of acute infection (clinical resolution/chronicity).

According to the EASL guidelines [8], patients with HBeAg-positive chronic infection had serum HBV-DNA > 7 logIU/mL and persistently normal serum alanine aminotransferase (ALT), while patients with HBeAg-positive chronic hepatitis were characterized by elevated serum HBV-DNA and ALT. Patients were classified in the HBeAg-positive chronic infection or hepatitis phases after a monitoring time of at least 6 months.

The upper limit of normal for ALT was set as 40 U/L, according to international guidelines for defining the disease phase [8].

Before starting immunosuppressive treatment, the 29 patients with HBV reactivation had a serological profile characterized by anti-HBc positivity and HBsAg negativity.

Immunosuppression-driven HBV reactivation was defined as a reappearance of serum HBV-DNA (>100 IU/mL) in patients positive to anti-HBc and negative to HBsAg during or after the administration of immunosuppressive therapy [11]. The most frequent (>10%) immunosuppressive conditions were related to a hematological disease (65.5%), an auto/chronic inflammatory disease (13.8%) and a solid organ transplantation (10.3%). Among the 29 patients, 15 were monitored every 3 months for at least 12 months after starting an antiviral therapy with entecavir or tenofovir (median (min–max) follow up: 21 (12–54) months). In particular, during monitoring, the following parameters were collected in order to assess the virological response to anti-HBV treatment: serum HBV-DNA, qualitative detection of HBeAg and transaminases. These parameters were used to define the percentage of patients achieving virological suppression (serum HBV-DNA < 20 IU/mL), ALT normalization (ALT < 40 U/L) and HBeAg loss (assessed by a qualitative assay, as per routine clinical practice). HBeAg quantification was carried out only in the plasma samples at the diagnosis of immunosuppression-driven HBV reactivation in order to establish the correlation with levels of HBsAg, serum HBV-DNA and ALT observed at the diagnosis of HBV reactivation and to evaluate the association with the achievement of HBeAg loss during anti-HBV treatment.

Ethics approval was deemed unnecessary because, under Italian law, biomedical research is subjected to previous approval by ethics committees only in the hypothesis of clinical trials on medicinal products for clinical use (art. 6 and art. 9, leg. decree 211/2003). The research was conducted on plasma samples (residual for clinical routine) and data that were previously anonymized, according to the requirements by Italian Data Protection Code (leg. decree 196/2003). Thus, all the plasma samples were already stored and not specifically collected for this study.

Data on demographics (sex, age, nationality), biochemical and virological parameters were collected and stored in an ad hoc designed anonymous database.

### 2.2. HBV Laboratory Evaluation

#### 2.2.1. Quantification of Serum HBV-DNA

Serum HBV-DNA was quantified by a real-time PCR system (COBAS Amplicor-COBAS TaqMan, Roche, Basel, Switzerland; lower limit of quantitation [LLoQ]:20 IU/m). The values of HBV-DNA were log transformed and expressed in log10 IU/mL.

#### 2.2.2. Quantification of HBsAg

Quantitative HBsAg (qHBsAg) was determined by the commercially available quantitative HBsAg detection system (Abbott Architect, lower limit of detection (LLoD): 50 mIU/mL (Abbott Diagnostics, Abbott Park, IL, USA).

#### 2.2.3. Quantification of HBeAg

HBeAg was quantified by DiaSorin LIAISON HBeAg chemiluminescent immunoassay (CLIA) with a linear range of 0.1–120 Paul-Ehrlich international (PEI) U/mL, characterized by a sensitivity and specificity of 99.5%, each (DiaSorin, Saluggia, Italy). Samples above 120 PEI U/mL were appropriately diluted to fall within the linear range of the assay.

Moreover, assay linearity was also confirmed by quantifying serially diluted plasma samples from 6 HBeAg-positive patients (Figure S1). The following dilutions were used: 1:10, 1:25, 1:50, 1:100, 1:250, 1:500, and 1:1000. Each dilution was carried out in duplicate by using the following diluents: DiaSorin Specimen Diluent and plasma from blood donors.

#### 2.2.4. Qualitative Detection of HBeAg and HBsAg

Qualitative HBeAg was determined by the commercially available ARCHITECT HBeAg (Abbott Architect, Abbott Park, IL, USA). Qualitative detection of HBeAg was used in patient’s monitoring during the follow-up to evaluate the HBeAg loss.

Qualitative HBsAg was determined by the commercially available assay (Abbott Architect, Abbott Park, IL, USA).

### 2.3. Statistical Analysis

Continuous variables were expressed as a median (interquartile range (IQR)) while discrete variables were expressed as absolute numbers or as percentages.

The statistically significant difference in qHBeAg between patients with HBeAg-positive hepatitis and the other three groups was assessed by the Mann–Whitney test.

The correlations of qHBeAg with serum HBV-DNA, qHBsAg and ALT were assessed by Spearman Rho test in each group of patients analyzed. *p*-values were corrected for multiple hypotheses testing by the Benjamini–Hochberg method (at a false discovery rate of 5%). Furthermore, median (IQR) qHBeAg was also evaluated, stratifying patients according to the levels of serum HBV-DNA. For this analysis, statistically significant differences were assessed by the Mann–Whitney test.

The Fisher exact test was used to determine the association of HBeAg and HBsAg levels (alone or in combination) with HBeAg loss during antiviral treatment in patients developing immunosuppression-driven HBV reactivation.

All the statistical analyses were carried out by GraphPad Prism version 8.0.1 for Windows (GraphPad Software, San Diego, CA, USA).

## 3. Results

### 3.1. Characteristics of the Study Population

This study included 101 HBeAg-positive patients: 20 with acute infection, 20 with HBeAg-positive chronic infection, 32 with HBeAg-positive chronic hepatitis and 29 with immunosuppression-driven HBV reactivation. The detailed characteristics of patients included in the different groups are reported in Table 1. Overall, patients were predominantly males (68.7%) with a median (IQR) age of 30 (24–54) years (Table 1). The continent of origin of most patients was Europe (54.1% Italy and 9.2% other European countries), followed by Asia (20.4%) and Africa (16.3%).

As expected, median serum HBV-DNA was elevated across all study groups (ranging from 6.8 log IU/mL in patients with reactivation up to 8.3 log IU/mL in acute infection), reflecting an intense viral replication, typical of HBeAg-positive phases (Table 1).

Similarly, high HBsAg levels were detected across all study groups, with the highest median (IQR) values observed in HBeAg-positive infection (64,673 [44,049–85,219] IU/mL) and the lowest in HBV-reactivation (31,882 [9902–52,000] IU/mL) (Table 1). Profound increases in ALT (>10× upper limit of normal [ULN]) were noted in most acute infections (median [IQR]: 1670 [183–2155] U/L), while less pronounced ALT flares (2–4× ULN) were observed in most patients with HBV reactivation and in those with HBeAg-positive chronic hepatitis (median [IQR]: 143 [40–528] U/L and 74 [52–137] U/L) (Table 1).

Overall, most patients were infected with HBV genotype D (44.2%), followed by genotype A (28.6%) and genotype E and F (9.1% for each) (Table 1).

### 3.2. HBeAg Levels across the Different HBeAg-Positive Phases of HBV Infection

**Figure 1:**
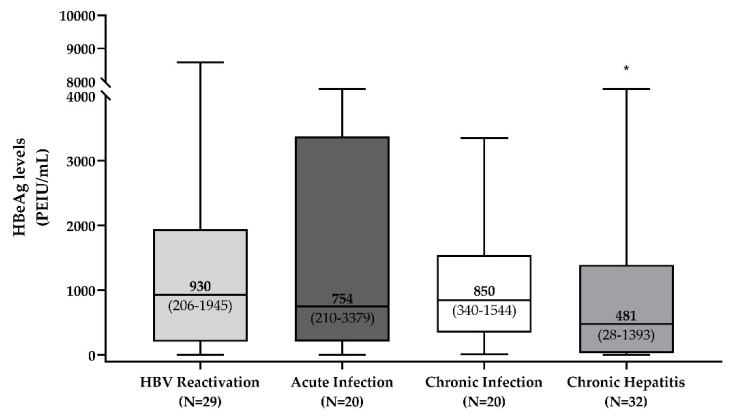
Distribution of HBeAg levels across different phases of HBV infection. The boxplot reports median, 25th and 75th percentile, min and max values of qHBeAg (expressed in PEIU/mL) in patients experiencing immunosuppression-driven HBV reactivation (N = 29), with acute infection (N = 20), with HBeAg-positive chronic infection (N = 20), with HBeAg-positive chronic hepatitis (N = 32). HBeAg was quantified by the CLIA Liaison HBeAg assay (DiaSorin). * Indicates the statistically significant difference (*p* = 0.03) observed by comparing HBeAg levels in patients with HBeAg-positive chronic hepatitis versus those in the other three groups. Statistically significant difference was assessed by the Mann–Whitney test.

According to the status of iatrogenic immunosuppression, median (IQR) qHBeAg tended to be higher in patients with HBV reactivation (median [IQR]: 930 [206–1945]) and slightly lower in patients with acute infection and with HBeAg-positive chronic infection (754 [210–3379] and 850 [340–1544] PEIU/mL, respectively). Conversely, in line with the development of an anti-HBV immune response, median (IQR) qHBeAg reached the lowest values in patients with HBeAg-positive chronic hepatitis (481 [28–1393] PEIU/mL, *p* = 0.03 compared to the other three groups) (Figure 1).

No statistically significant differences were noted in median (IQR) qHBeAg across the different HBV genotypes and according to the demographic characteristics.

### 3.3. Correlation of HBeAg Levels with the Other HBV Biomarkers

The correlation of HBeAg levels with serum HBV-DNA, qHBsAg and ALT was then evaluated in each group of patients.

#### 3.3.1. Acute Infection

**Figure 2:**
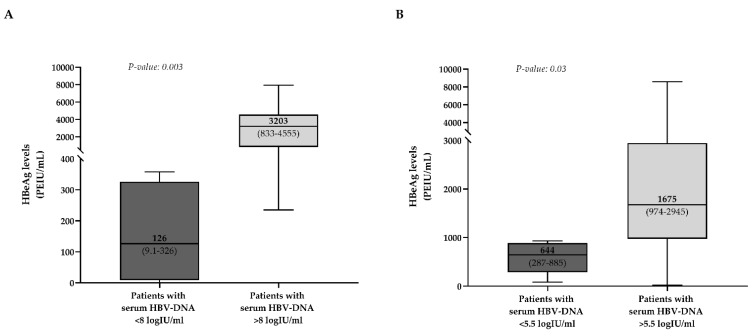
Distribution of HBeAg levels in patients stratified according to serum HBV-DNA in patients with acute HBV infection and immunosuppression-driven HBV reactivation. Boxplots report median, 25th and 75th percentile, min and max values of qHBeAg (expressed in PEIU/mL) in patients with acute infection (**A**) stratified according to serum HBV-DNA lower or higher than 8 logIU/mL and in patients experiencing immunosuppression-driven HBV reactivation (**B**) stratified according to serum HBV-DNA lower or higher than 5.5 logIU/mL. Statistically significant differences were assessed by the Mann–Whitney test.

In acute infection, qHBeAg showed a strong positive correlation with qHBsAg (Rho = 0.78, *p* = 0.0006) and a negative correlation with ALT (Rho = −0.66, *p* = 0.006) (Table 2). A negative correlation was also observed between qHBsAg and ALT (Rho = −0.63, *p* = 0.03) (Table S1). Furthermore, even if a significant correlation was not observed between qHBeAg and serum HBV-DNA, a higher qHBeAg characterized patients with serum HBV-DNA > 8 logIU/mL (median [IQR]: 3203 [833–4555] vs. 126 [9–326] PEIU/mL, *p* = 0.003) (Figure 2A).

Among patients with acute infection, three did not achieve HBsAg loss after at least 12 months of monitoring, thus developing chronicity. Notably, these three patients had a higher median (IQR) qHBeAg than those achieving HBsAg loss (clinical resolution of HBV infection) (2555 [1365–2555] vs. 299 [189–3203] PEIU/mL).

#### 3.3.2. HBeAg-Positive Chronic Infection and Hepatitis

In HBeAg-positive chronic infection, qHBeAg showed no correlation with qHBsAg and serum HBV-DNA. The only significant correlation was noted between qHBsAg and serum HBV-DNA (rho = 0.56, *p* = 0.013) (Table S1).

A different correlation profile was observed in HBeAg-positive chronic hepatitis. Indeed, in this group of patients, qHBeAg was positively correlated with both qHBsAg (rho = 0.62, *p* < 0.001) and serum HBV-DNA (rho = 0.57, *p* = 0.0008) (Table 2). A positive correlation was also observed between qHBsAg and serum HBV-DNA (rho = 0.63, *p* = 0.0002) (Table S1). Furthermore, in HBeAg-positive chronic hepatitis, qHBeAg was the only biomarker tending to negatively correlate with ALT (Rho = −0.35, *p* = 0.05 for qHBeAg; rho = −0.23, *p* = 0.21 for qHBsAg; rho = −0.12, *p* = 0.51 for serum HBV-DNA) (Table 2 and S1).

#### 3.3.3. Immunosuppression-Driven HBV Reactivation

In patients developing immunosuppression-driven HBV-reactivation, qHBeAg was positively correlated with qHBsAg (rho = 0.59, *p* = 0.007) (Table 2). A positive correlation was also observed between qHBsAg and serum HBV-DNA (rho = 0.67, *p* = 0.002) (Table S1). Even if a significant correlation was not observed between qHBeAg and serum HBV-DNA, a higher qHBeAg was observed in patients with serum HBV-DNA > 5.5 logIU/mL (median [IQR]: 1675 [974–2945] vs. 644 [286–885] PEIU/mL, *p* = 0.03) (Figure 2B).

**Figure 3:**
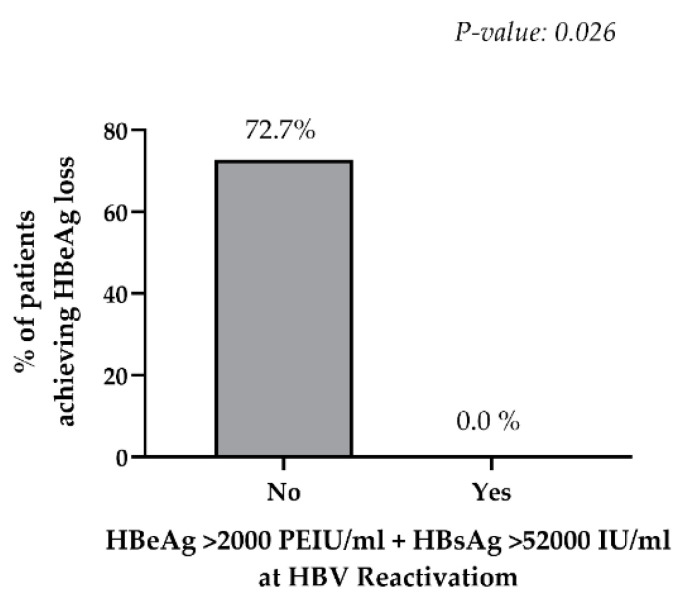
Percentage of patients achieving HBeAg loss. The histogram reports the percentage of patients achieving the HBeAg loss after starting anti-HBV treatment in patients without qHBeAg > 2000 PEIU/mL + qHBsAg > 52,000 IU/mL (grey bar) and in patients with this combination at HBV-reactivation. Statistically significant differences were assessed by the Fisher exact test.

As a final step, the association of qHBeAg with HBeAg loss in patients experiencing HBV-reactivation under iatrogenic immunosuppression was evaluated. In particular, among the 29 patients with HBV reactivation, 15 were monitored for at least 12 months after starting an antiviral therapy with entecavir or tenofovir (median [min-max] follow up: 21 [12–54] months). ALT normalization was achieved by 93% of patients, while HBeAg loss and serum HBV-DNA suppression was observed in 53.3% and 60% of patients, respectively. Notably, based on the data, the combination of qHBeAg > 2000 PEIU/mL and qHBsAg > 52,000 IU/mL was the best and the only cut-off, maximizing the probability of not achieving HBeAg loss during anti-HBV treatment. Indeed, no patients with this specific combination (0/4) achieved HBeAg loss versus 72.7% (8/11) of patients without it (*p* = 0.026) (Figure 3), despite a comparable follow-up time (median [IQR] months 23 [12–33] in patients with and 21 [12–38] in patients without qHBeAg > 2000 PIEU/mL and qHBsAg > 52,000 IU/mL). No statistical significance was reached when qHBeAg and qHBsAg were analyzed separately (25% vs. 70%, P = 0.12 for qHBeAg and 40% vs. 62.5%, *p* = 0.59 for qHBsAg).

## 4. Discussion

This is one of the first studies evaluating the levels of HBeAg across different stages of HBV infection and their correlation with virological and biochemical biomarkers. In particular, the highest median HBeAg levels were observed in patients undergoing immunosuppression-driven HBV reactivation while the lowest levels were reached in patients with HBeAg-positive chronic hepatitis. This result can be explained by the elevated replicative activity coupled with a deficient immune response against the virus, characterizing the status of immunosuppression-driven HBV reactivation. Conversely, the lowest median HBeAg levels were observed in patients with HBeAg-positive chronic hepatitis. Different from the other three groups, HBeAg-positive hepatitis is also defined as an immune-active phase, characterized by substantial liver necroinflammation that progressively reduces the pool of infected hepatocytes and in turn HBV replicative activity [9,10]. In the long term, this can lead to HBeAg loss, which is usually considered a clinically relevant endpoint associated with minimal risk of developing end-stage liver diseases [9,10]. In this light, the lowest qHBeAg, observed in this phase, can reflect the attempt of the immune system to constrain the extent of the intrahepatic HBV reservoir and of viral replication, critical events preceding HBeAg loss.

By analyzing the correlations among virological markers, qHBeAg showed positive correlations with qHBsAg across all study groups (with the exception of HBeAg-positive chronic infection), suggesting that HBeAg production parallels that of HBsAg. In particular, it is plausible that in the setting of HBeAg positivity, the synthesis of both HBeAg and HBsAg is mainly sustained by the transcriptional activity of cccDNA with a limited contribution of integrated HBV-DNA to the HBsAg production. This is in line with different studies supporting that integrated HBV-DNA can represent a major source of HBsAg, mostly in HBeAg-negative patients [22,23].

Conversely, weaker correlations were noted between qHBeAg and serum HBV-DNA. As also suggested by Thompson and coworkers [24], this result may reflect a potential “*disconnection”* between HBeAg production pathway and the viral replication pathway, including additional steps such the encapsidation of the pre-genomic RNA followed by the reverse transcription process.

Notably, qHBeAg was the biomarker showing the strongest negative correlation with ALT, specifically in the setting of acute infection and chronic hepatitis. This result is in keeping with a recent study showing an inverse correlation of HBeAg levels with ALT and APRI scores in patients with chronic HBV hepatitis [25]. It is known that HBeAg can act as a tolerogen and immunomodulator, thus jeopardizing the establishment of a fully effective immune response against the virus [5]. Based on this assumption, lower HBeAg levels can reflect a waning tolerogenic activity that parallels the development of a cytotoxic immune response, critical to achieve the clinical resolution of HBV infection or entry into the HBeAg-negative chronic infection. In acute infection, this is also supported by the fact that patients achieving the clinical resolution of infection tend to have lower HBeAg levels than those developing chronicity.

These results advocate setting up longitudinal studies to unravel the interplay between HBeAg kinetics and the extent of immunological pressure and to establish the role of qHBeAg as biomarker to assess not only the extent of HBV replicative activity, but also the development of cytolytic activity against HBV.

However, in a small set of patients, this study has also shown that the combination of qHBeAg > 2000 PEIU/mL + qHBsAg > 52,000 IU/mL (presumably reflecting an extensive intrahepatic HBV transcriptional activity) at HBV reactivation is significantly associated with no HBeAg loss during anti-HBV therapy in the setting of iatrogenic immunosuppression. It should be stressed that this is an exploratory analysis that requires confirmation in a larger sample size in order to validate the above-mentioned cut-off of biomarkers and to improve the generalizability of the result obtained.

Nevertheless, this finding is in keeping with a previous study led in HIV + HBV co-infected patients initiating an HBV-active antiretroviral regimen, showing that low levels of HBsAg and HBeAg are significantly correlated with HBeAg seroconversion [26].

The overall findings suggest the advantage of qHBeAg (alone or in combination with other virological biomarkers) as a potential predictive marker of treatment outcome in HBeAg-positive patients. On these bases, further longitudinal studies on larger datasets of patients are necessary to better clarify the role of HBeAg quantification in predicting the response to anti-HBV treatment, particularly in the setting of a weakened immune response.

We acknowledge that the sample size of this study is relatively small. This can be explained by the fact that in Europe, most HBV-infected patients are HBeAg negative and by the exclusion of patients coinfected with HIV, HCV or HDV. Furthermore, another limiting factor was the need for a sufficient amount of plasma for the quantification of HBeAg and (when necessary) of other HBV markers of replication.

In conclusion, HBeAg levels differ over the natural course of HBV infection and according to the extent of immunological pressure and cytolytic activity against the virus. In the setting of iatrogenic immunosuppression, HBeAg levels are associated with a virological response to anti-HBV therapy.

## Figures and Tables

**Table 1:** Patients’ characteristics.

	Overall (N = 101)	Acute Infection (N = 20)	eAg+ Chronic Infection (N = 20)	eAg+ Chronic Hepatitis (N = 32)	HBV Reactivation (N = 29)
Age, years, median (IQR)	30 (24–54)	40 (24–43)	25 (21–27)	29 (19–38)	59 (49–72)
Male, N (%)	68 (68.7)	19 (95.0)	9 (45.0)	22 (73.3)	18 (62.1)
Italian nationality, N (%)	53 (54.1)	17 (85.0)	1 (5.0)	9 (28.1)	26 (89.7)
Serum HBV-DNA, log IU/mL, median (IQR)	8.1 (6.8–8.4)	8.3 (7.9–8.7)	8.2 (8.0–8.5)	8.2 (7.1–8.6)	6.8 (5.6–8.0)
HBsAg levels, IU/mL, median (IQR)	48,000 (12,000–60,829)	48,000 (12,500–52,000)	64,673 (44,049–85,219)	35,270 (10,338–65,236)	31,882 (9902–52,000)
ALT, U/L, median (IQR)	71 (39–227)	1670 (183–2155)	29 (21–35)	74 (52–137)	143 (40–528)
ALT, N × ULN ^1^	1.8× (1.0×–5.7×)	41.8× (4.6×–53.9×)	0.7× (0.5×–0.9×)	1.9× (1.3×–3.4×)	3.6× (1.0×–13.2×)
HBV-Genotypes, N (%)					
D	34 (44.2)	4 (23.5)	2 (28.6)	7 (50.0)	21 (72.4)
A	22 (28.6)	8 (47.1)	1 (14.3)	7 (50.0)	6 (20.7)
E	7 (9.1)	0 (0.0)	1 (14.3)	6 (42.9)	0 (0.0)
B	4 (5.2)	0 (0.0)	3 (42.9)	1 (7.1)	0 (0.0)
F	7 (9.1)	5 (29.4)	0 (0.0)	1 (7.1)	1 (3.4)
C	2 (2.6)	0 (0.0)	0 (0.0)	2 (14.3)	0 (0.0)
G	1 (1.3)	0 (0.0)	0 (0.0)	0 (0.0)	1 (3.4)
Not available	25 (32.5)	3 (15.0)	13 (65.0)	8 (25.0)	0 (0.0)

^1^ N × ULN (upper limit normal): times of ALT increase respect to normal value of 40 U/L, according to EASL guidelines [8].

**Table 2:** Correlation of HBeAg levels with virological and biochemicals markers.

		Correlation of HBeAg Levels with		
		HBsAg Levels	Serum HBV-DNA	ALT Levels
Acute infection	*rho* *^a^*	0.78	0.27	−0.66
	*p-value* *^a^*	0.0006	0.9	0.006
Chronic infection	*rho* *^a^*	0.04	0.23	−0.35
	*p-value* *^a^*	0.9	0.3	0.9
Chronic hepatitis	*rho* *^a^*	0.62	0.57	−0.35
	*p-value* *^a^*	0.0002	0.0008	0.05
HBV-reactivation	*rho* *^a^*	0.59	0.17	0.04
	*p-value* *^a^*	0.007	0.5	0.9

*^a^* Correlations were assessed by Spearman’s rho test. Statistically significant correlations are in bold. *p*-values remaining significant after correction for multiple hypotheses (by the Benjamini–Hochberg method at a false discovery rate of 5%) are reported as underlined.

## Data Availability

The data that support the findings of this study are available from the corresponding author upon reasonable request.

## Supplementary Materials

The following are available online at https://www.mdpi.com/article/10.3390/biomedicines9101352/s1, Figure S1: Linearity of the assay, Table S1: Correlation of virological and biochemical parameters.
