# Supplementary material for: HBeAg Levels Vary across the Different Stages of HBV Infection According to the Extent of Immunological Pressure and Are Associated with Therapeutic Outcome in the Setting of Immunosuppression-Driven HBV Reactivation
Source: Biomedicines. 2021 Sep 29;9(10):1352. doi: 10.3390/biomedicines9101352 (PMC8533134; doi:10.3390/biomedicines9101352)
Supplement: Supplementary file 1 [file biomedicines-09-01352-s001.zip › biomedicines-1350277-supplementary.pdf]

Supplementary Materials

Table S1. Correlation of virological and biochemical parameters

		Correlation of qHBsAg with		Correlation of serum HBV-DNA with ALT
		**Serum HBV-DNA**	**ALT**	
**Acute infection**	*rho*[a]	0.27	**-0.63**	-0.28
	P-value[a]	0.34	**0.03**	0.31
**Chronic infection**	*rho*[a]	**0.56**	-0.04	-0.19
	P-value[a]	**0.01**	0.89	0.44
**Chronic hepatitis**	*rho*[a]	**0.63**	-0.23	-0.12
	P-value[a]	**0.0002**	0.21	0.51
**HBV reactivation**	*rho*[a]	**0.67**	-0.13	0.08
	P-value[a]	**0.002**	0.64	0.74

[a] Correlations were assessed by Spearman's rho test. Statistically significant correlations are in bold. P-values remaining significant after correction for multiple hypotheses (by Benjamini-Hochberg method at false discovery rate of 5%) are reported as underlined.

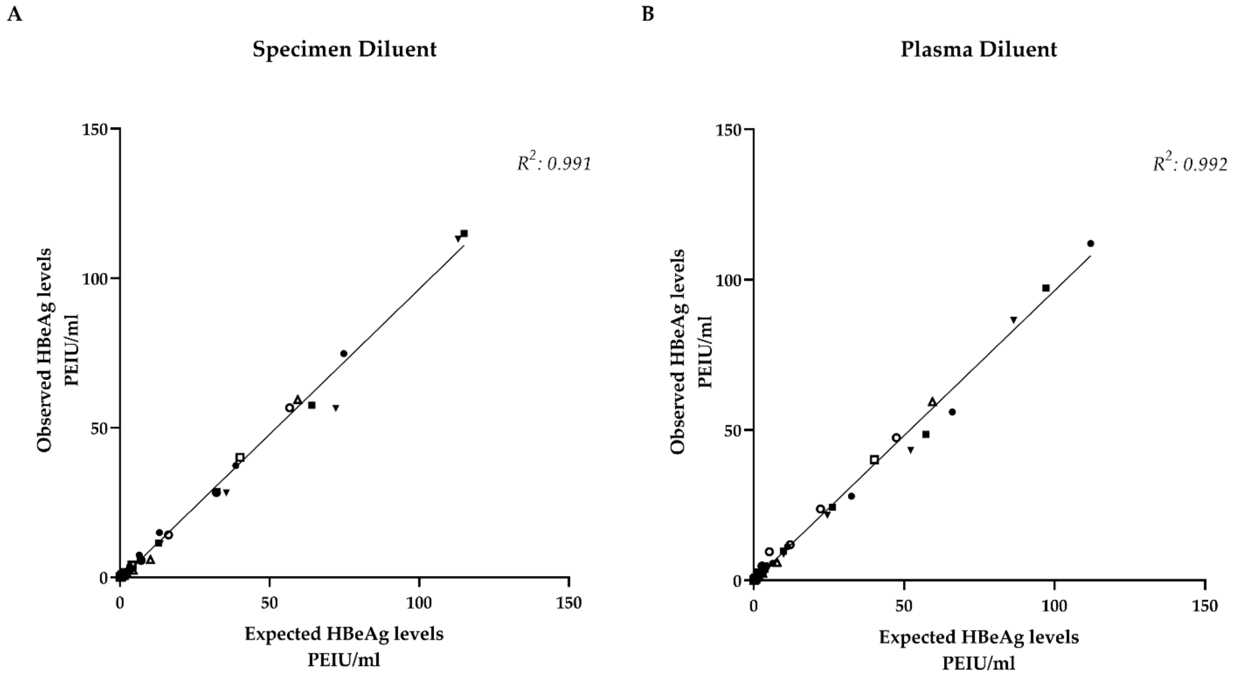

Figure S1. Linearity of the assay. The graph in panel A reports the linearity of the assay obtained from serial dilutions of 6 sample made by DiaSorin Specimen Diluent. The panel B shows the serial dilutions of the same samples obtained using plasma from a blood donor.
